# Xuming Zhusan Decoction Attenuates Post‐Stroke via Modulating TLR4/MYD88/NF‐κB Pathway in Mice

**DOI:** 10.1002/fsn3.71734

**Published:** 2026-04-16

**Authors:** Xi Liu, Cheng Wang, Ling Han, Yi'an Xiao, Hanchang Yu, Zhongxu Hu, Xiyun Fei, Min Peng, Jilin Zhou, Zhijun Zhong

**Affiliations:** ^1^ Department of Neurosurgery Changsha Hospital of Traditional Chinese Medicine (The Eighth Hospital of Changsha) Changsha Hunan China

**Keywords:** ischemic stroke, microglial, neuroinflammation, neurological function, TLR4/MYD88/NF‐κB, Xuming Zhusan (XMZS)

## Abstract

Ischemic stroke induces neuroinflammation that exacerbates neuronal damage. The Chinese Medicine Xuming Zhusan (XMZS) has shown promising efficacy in stroke treatment, but its mechanisms are unclear. This study aimed to explore the effect of XMZS on microglial‐mediated neuroinflammation and explore its molecular targets. In vivo experiments were conducted using a mouse model of cerebral ischemia–reperfusion injury induced by middle cerebral artery occlusion (MCAO), and in vitro studies were performed on primary microglia and cortical neurons with Oxygen–Glucose Deprivation/Reoxygenation (OGD/R) models. Behavioral assessments, histological analyses, Western blotting, co‐immunoprecipitation, confocal microscopy, and enzyme‐linked immunosorbent assay (ELISA) were employed to evaluate the neuroprotective effects of XMZS and its regulatory role in the TLR4/MYD88/NF‐κB pathway. Results showed that high‐dose (63 g/kg) XMZS improved neurological and cognitive functions, reduced infarct volume and brain edema in MCAO mice. Mechanistically, XMZS suppressed microglial activation, decreased the levels of pro‐inflammatory cytokines (IL‐1β, IL‐6, TNF‐α), and downregulated the expression of TLR4, MYD88, and phosphorylated p65 in the TLR4/MYD88/NF‐κB pathway. Additionally, cinditioned medium from XMZS‐treated microglia enhanced the viability of primary neurons. In conclusion, XMZS exerts neuroprotective effects against cerebral ischemia–reperfusion injury by modulating the TLR4/MYD88/NF‐κB pathway, thereby attenuating microglia‐mediated neuroinflammation.

## Graphical abstract

High‐dose Xuming Zhusan (XMZS) improves neurological/cognitive functions and reduces cerebral infarct in MCAO mice. It inhibits microglial activation and pro‐inflammatory cytokines via suppressing the TLR4/MYD88/NF‐κB pathway, protecting neurons.

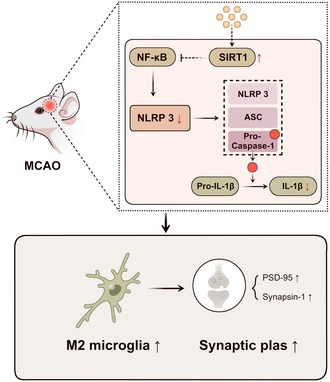

## 1 Introduction

Stroke is intricately linked to the global health crisis. In 2019 alone. There were 122 million incident cases of stroke worldwide, along with 101 million prevalent cases, and 6.55 million deaths attributed to stroke (GBD 2019 Stroke Collaborators 2021). The 2017 Global Burden of Disease (GBD) analysis indicated that while age‐standardized mortality rates for stroke declined significantly between 1990 and 2017, the reduction in age‐standardized incidence was far less pronounced. This implies that the preventive strategies have been less effective than therapeutic interventions (Chen et al. 2023). Ischemic stroke accounts for 85% of all strokes and triggers a cascading neuroinflammatory response that exacerbates neuronal damage and determines long‐term prognosis (Lombardozzi et al. 2025). Following cerebral ischemia, damaged neurons and glia cells release damage‐associated molecular patterns, activating microglia and recruiting peripheral leukocytes. These cells collectively secrete pro‐inflammatory cytokines (e.g., TNF‐α, IL‐1β) and matrix metalloproteinases (MMPs) (Olivera et al. 2018). Although acute inflammation plays a crucial role in clearing away the remnants, the sustained activation of microglia within the ischemic penumbra leads to neurotoxicity, the breakdown of the blood–brain barrier (BBB), and the delayed death of neurons (Peng et al. 2022). These negative consequences not only exacerbate the damage to the nervous system but also pose significant challenges to the recovery and prognosis of patients suffering from ischemic‐related brain injuries, highlighting the importance of precise regulation of the inflammatory response in clinical treatment.

Microglia play a pivotal role in determining the outcomes of neuro‐inflammation. During ischemic injury, these cells polarize into pro‐inflammatory M1 phenotypes, characterized by upregulation of TLR4/MYD88/NF‐κB signaling pathway and production of reactive oxygen species (ROS), which results in amplifying tissue damage (Li et al. 2022; Yang et al. 2020). Conversely, anti‐inflammatory M2 phenotypes promote repair by secreting neurotrophic factors (such as TGF‐β) and resolving inflammation (Liu et al. 2023; Bodhankar et al. 2015). Dysregulation of this M1/M2 balance is associated with larger infarct volumes, more severe neurological deficits, and increased risk of post‐stroke depression (Nagy et al. 2020). As a key driver of microglial activation, the TLR4/MYD88/NF‐κB pathway integrates signals from damage‐associated molecular patterns to induce proinflammatory gene transcription, making it a promising therapeutic target (Su et al. 2018). Previous studies have shown that TLR4 inhibition reduced infarct size by 30%–40% and improved motor function recovery in mouse models of ischemic stroke (Hua et al. 2007). Another study has found that the 5‐lipoxygenase inhibitor could reduce the cerebral infarction volume in rats from 512.2 mm^3^ to 215 mm^3^, and this inhibitor exerted its effect by inhibiting the activation of NF‐κB (Jatana et al. 2006). In summary, understanding the complex interplay among microglial phenotypes, the TLR4/MYD88/NF‐κB pathway, and ischemic injury outcomes is crucial for developing novel and effective neuroprotective strategies against ischemic stroke.

Traditional Chinese medicine (TCM) provides unique therapeutic strategies for various diseases globally. Among them, Xuming Zhusan (XMZS), a formula from *Emergency Prescriptions Worth a Thousand Gold* (Tang Dynasty, Sun Simiao), is of interest for stroke treatment. It exerts multi‐targeted actions, such as exterior‐relieving and pathogen‐clearing, promoting blood circulation and resolving stasis, orifice‐opening and resuscitation‐inducing (Tang et al. 2008).

Though XMZS shows clinical benefits for stroke, its molecular mechanism, especially its effects on post‐stroke microglial neuroinflammation, remain unclear. In this study, based on TCM theory and prior research, we aim to comprehensively investigate how XMZS exerts its effects on microglial‐mediated neuroinflammation, with the hope of uncovering novel insights into its therapeutic actions and providing a scientific basis for its broader clinical application in treating stroke and related neurological conditions.

## 2 Materials and Methods

### 2.1 Preparation of XMZS

All herbal materials of XMZS (listed in Table S1) were purchased from Shandong Bencao Pharmaceutical Co. Ltd. (Zibo, China) and identified to conform to the quality standards specified in the Chinese Pharmacopeia (2022 Edition). The herbal formula XMZS (batch number: XMZS‐Lab‐20240402) was prepared in our laboratory by boiling 12 herbs together; in brief, *Pueraria lobata* (30 g), *Heracleum hemsleyanum* Diels (15 g), *Paeonia lactiflora* Pall. (15 g), *Angelica sinensis* (15 g), *Cinnamomum cassia* Presl (12 g), *Polygala tenuifolia* Willd. (12 g), Radix *Ginseng* (10 g), *Saposhnikovia divaricata* (10 g), *Rehmannia glutinosa* Libosch. (10 g), *Ligusticum chuanxiong* (9 g), *Ephedra sinica* Stapf (9 g), *Asarum heterotropoides* F. Schmidt (6 g), and *Nepeta cataria* (10 g).

In brief, a total of 100 g of the combined herbal mixture was added to 500 mL of distilled water. The mixture was then boiled for 40 min. Subsequently, the decoction was carefully filtered, and the liquid was collected. The remaining herbal residues were further boiled in 300 mL of distilled water for another 40 min. After this second boiling, the resulting decoction was also filtered and collected. The two portions of the decoction obtained from these processes were then blended together, and were concentrated and stored at 4°C for further use in this experiment.

For quality control, the extraction of the XMZS decoction was monitored, and the content of key active components was determined by high‐performance liquid chromatography (HPLC) to ensure the stability of the drug.

### 2.2 Animals Rearing and Grouping

A total of 100 C57BL/6 mice (8–10‐week‐old, weighing 25 ± 2 g) were obtained from Bowei Biotechnology Co. Ltd. in China. These mice were housed under controlled conditions (temperature: 22°C; 12‐h light/dark cycle) with free access to standard chow and water. The animal research protocol was approved by the Animal Welfare Ethics Committee of Beijing MDKN Biotechnology Co. Ltd. (approval number: MDKN‐2024‐041).

Mice were randomly divided into five groups (20 per group). (1) Sham group: subjected to sham surgery without middle cerebral arteries occlusion; (2) Model group: established as the ischemic strole model; (3) MCAO‐L group: MCAO mice treated with low‐dose XMZS (31.5 g/kg/day); (4) MCAO‐H group: MCAO mice treated with high‐dose XMZS (63 g/kg/day); and (5) MCAO + AAV‐TLR4 + XMZS‐H group: MCAO mice injected with AAV‐TLR4 (for TLR4 overexpression) and treated with high dose XMZS (63 g/kg/day) starting from Day 3 post‐MCAO. XMZS was administered via gavage for 14 consecutive days, with dosages referenced from previous studies (Chen et al. 2023).

### 2.3 MCAO Procedure

The MCAO procedure was performed as previously described (GBD 2019 Stroke Collaborators 2021). Mice were anesthetized with 3% isoflurane in a mixture of 70% N_2_O and 30% O_2_ and maintained with 1.5%–2.0% isoflurane during surgery. Body temperature was kept at 37°C ± 0.5°C using a heated pad. After shaving and disinfecting the neck area, a midline incision was made to expose the common carotid artery. A 4–0 nylon suture with silicone (from Shandong Boda Medical products Co. Ltd., China) was inserted through the left internal carotid artery until resistance was felt, occluding the vessel for 60 min. The suture was then removed to allow reperfusion, and the neck wound was closed. Intraoperative fluid loss was compensated with 1.0 mL of normal saline. Mice were allowed to recover on a heated pad for 24 h, with food and water provided ad libitum 12 h after ischemia induction.

### 2.4 AAV‐TLR4 Overexpression via Stereotaxic Injection

Seven days prior to MCAO surgery, mice were anesthetized with 2% isoflurane fixed on a stereotaxic frame. AAV9‐TLR4 virus (titer: 1 × 10^12^ vg/mL, PackGene Biotech, Guangzhou, China) was stereotaxically injected into the right striatum (AP +0.5 mm, ML +2.0 mm, DV −3.0 mm) at a rate of 0.2 μL/min (total volume: 2 μL), followed by a 10‐min post‐injection dwell time. Buprenorphine (0.05 mg/kg, subcutaneous injection) was given for analgesia during the 7‐day feeding period to ensure viral expression. Mice then underwent MCAO surgery or Sham surgery as described above. Three days after surgery, mice in the MCAO + AAV‐TLR4 + XMZS‐H group received high‐dose XMZS (63 g/kg) via gavage. Behavioral tests were performed on 7 days post‐MCAO, and mice were euthanized on Day 14 for brain tissue collection and molecular analyses.

### 2.5 Modified Neurological Severity Score (mNSS) Test

Evaluated on Days 1, 3, 7, and 14 post‐reperfusion. The test includes motor, balance, sensory, reflex, and corneal reflex assessments, with a scoring range of 0 (normal) to 18 (severe deficits).

### 2.6 Adhesive Removal Test

Adhesive removal test was conducted on Days 1, 3, 7, and 14 post‐reperfusion to assess forepaw sensitivity and motor function. Mice were habituated for 3 days prior to testing. Circular adhesive stickers (4 × 3 mm) were applied to the mid‐paw area of both forelimbs, and the time to the first contact and removal of the stickers was recorded (maximum: 120 s). Each mouse was tested 3 times per day, with the average value used for analysis.

### 2.7 Corner Test: Ipsilateral Turning

The corner test was carried out on Day 1, 3, 7, and 14, after MCAO surgery. Mice were placed in a 30° corner formed by two rectangular boards (30 × 20 cm) and observed for ipsilateral turns. The percentage of ipsilateral turns out of 10 trials was calculated.

### 2.8 Novel Object Recognition Test

The novel objective recognition test was performed to assess cognitive function. Mice were habituated in a gray organic glass box (40 × 40 cm) for 10 min, followed by a 15 min familiarization phase with two identical objects. 30 min later, one object was replaced with a novolone, and exploration time for each object was recorded for 15 min. The recognition index (RI) was calculated as RI = (exploration time of new object/exploration time of familiar object) × 100%.

### 2.9 Infarct Volume Measurement by TTC Staining

Mice were euthanized on Day 7 post MCAO. Brains were removed, frozen at −20°C for 15 min, and cut into 2‐mm coronal sections. Sections were incubated in 2% 2,3,5‐triphenyltetrazolium chloride (TTC) (Sinopharm Chmical Reagent Co. Ltd., China) at 37°C for 20 min in the dark, then fixed with 4% formaldehyde (Shanghai Yubo Biotechnology Co. Ltd., China) overnight at 4°C. Infarct volume was quantified using Image‐Pro Plus 6.0 software (Media Cybernetics, USA), and expressed as a percentage of the contralateral hemispheres' volume.

For measurement of brain water content, brains were removed immediately after euthanasia, and wet weight was measured. Brains were then dried at 105°C for 48 h to determine dry weight. Brain water content was calculated according to the formula: brain water content (%) = (wet weight − dry weight)/wet weight × 100%.

### 2.10 Hematoxylin–Eosin (HE) Staining for Detection of Brain Injury and Nissl Staining for Observation of Neurons Protection

Brain tissues were fixed in 4% paraformaldehyde for 24 h, dehydrated, embedded in paraffin, and cut into 5 μm sections. Slides were deparaffinized, hydrated, and then stained with HE and observed under a 20× microscope to evaluate tissue change.

For Nissl staining, paraffin sections were hydrated, incubated in 0.1% toluidine blue at 37°C for 30 min, dehydrated, and observed under a light microscope to assess neuronal survival in the cortex, hippocampal CA1, and CA3 regions.

### 2.11 Immunofluorescence Analysis

Brain tissues were fixed with 4% formaldehyde for 2 h, cryoprotected in 30% sucrose for 48 h, embedded in optimal cutting temperature compound, and cut into 20‐μm‐thick sections. Sections were blocked with 5% bovine serum albumin (BSA) in PBS for 1 h at room temperature.

For single‐staining experiments targeting Iba1 and NeuN, primary antibodies against Iba1 (1:1000, Wako) and NeuN (1:1000, Abcam, UK) were incubated at 4°C for 15 h, followed by fluorescent secondary antibodies.

For double‐staining, primary antibodies against TLR4 (1:100, Abcam, UK) and p‐p65 (1:200, Abcam) were co‐incubated at 4°C overnight, followed by Alexa Fluor 594‐conjugated and Alex Fluor 448‐conjugated secondary antibodies (1:2000, Abcam) for 1–2 h in the dark.

Sections were counterstained with DAPI to visualize cell nuclei and imaged using an Olympus IX83 inverted fluorescence microscope. Iba1‐positive cells were quantified using Image J software (version 1.53c, NIH), with circularity, average branch length, and branch number analyzed.

### 2.12 Western Blot Analysis

Total proteins were extracted from mouse brain samples using Radio Immunoprecipitation Assay (RIPA) lysis buffer containing Phenylmethylsulfonyl Fluoride (PMSF), and protein concentrations were determined by the BCA method. Samples were mixed with 5 × sodium Dodecyl Sulfate (SDS) loading buffer, denatured at 100°C, and then cooled.

After Sodium Dodecyl Sulfate‐Polyacrylamide Gel Electrophoresis (SDS‐PAGE), proteins were transferred to Polyvinylidene Fluoride (PVDF) membranes, which were blocked with 5% non‐fat milk at room temperature for 2 h. Membranes were then incubated overnight at 4°C with primary antibodies against TLR4 (1:500, Abcam), MYD88 (1:500, Abcam), p65 (1:1000, Abcam), p‐p65 (p‐p65, 1:500, Abcam), and β‐actin (1:1000, Abcam) as internal control. Next day, membranes were washed with Tris‐Buffered Saline with Tween‐20 (TBST), and incubated with horseradish peroxidase (HRP)‐conjugated goat anti‐rabbit IgG (1:2000, Abcam) at room temperature for 1 h. After re‐washing, enhanced chemiluminescence (ECL) reagents were applied for protein bands imaging. Band intensities were analyzed via ImageJ, and relative target protein expression was calculated with β‐actin for normalization.

### 2.13 Detection of IL‐1β, IL‐6, and TNF‐α by ELISA

To explore the function of inflammatory factors in stroke progression, IL‐1β, IL‐6, and TNF‐α levels were detected using an ELISA kit (ColorfulGene BioTech. Co. Ltd., Wuhan, China). After incubation, washing, and color development, absorbance at 450 nm was measured by microplate reader. Cytokine contents were calculated from standard curve, and expressed as pg/mg protein.

### 2.14 Primary Microglial Cell Isolation

The method was adapted from Gordon et al. (Gordon et al. 2011; Olivera et al. 2018). First, 10‐day‐old mice were sacrificed, brains were aseptically removed and enzymatically digested in Dulbecco's Modified Eagle Medium (DMEM) supplemented with 10% fetal bovine serum (FBS) and 1% penicillin–streptomycin, with gentle agitation at 4°C. Cell suspensions were filtered through 70‐μm strainers, counted, and seeded into T75 flasks at 3 × 10^6^ cells/flask in microglia‐specific medium. After 7 days of confluent glial layer formation, flasks were shaken at 200 rpm for 3 h at 37°C. Supernatants were centrifuged at 1000 rpm for 5 min, pellets resuspended in DPBS with 0.5% BSA and 2 nM EDTA. CD11b microbeads (10 beads per cell) were added, incubated at 4°C for 30 min with shaking. CD11b‐positive microglia were isolated via magnetic separator, washed three times, resuspended in the specific medium, and seeded onto coverslips in 24‐well plates. Microglial purity was confirmed by immunostaining.

### 2.15 Oxygen–Glucose Deprivation/Reoxygenation (OGD/R) Model Establishment and Drug Intervention

Isolated and purified microglia cells were placed in a glucose‐free medium and then incubated in an anaerobic incubator for 6 h to simulate ischemic‐hypoxia, then transferred to a normal medium for reperfusion. Model success was verified by microglial morphological and functional changes and biomarker detection.

XMZS‐containing serum (5%, 10%, 15%, 20%, 30%, 40%, 50%) was added to the cell culture system. 10% concentration was selected via CCK‐8 assay for optional effect. The cells were divided into different groups: Control group: No OGD/R, no drug; OGD/R group: ischemia–reperfusion only; OGD/*R* + MS‐10% + OE‐TLR4 NC group: 10% medicated serum + TLR4 overexpression negative control; OGD/*R* + MS‐10% + OE‐TLR4 group: 10% medicated serum + TLR4 overexpression.

### 2.16 Observation of Protein Expression and Localization by Confocal Microscopy

Immunofluorescence staining and confocal microscopy were performed to explore MS‐10% effects on microglial protein expression. Procedures followed antibody incubation, fluorescent labeling, and imaging as in animal experiments, with additional detection of iNOS. Primary anti‐iNOS antibody (1:1000, ab15323, Abcam) was incubated at 2 μg/mL, followed by goat anti‐rabbit IgG (1:2000, Abcam) under the same conditions as other proteins.

### 2.17 Isolation and Identification of Cortex Neurons

Mouse cerebral cortex tissues were dissected, minced, and digested in 0.125% trypsin (Biolad, Technology, Beijing, China) at 37°C for 15 min. Dissociated neurons were collected by centrifugation and cultured in conditioned media from different microglia groups at 37°C, 5% CO_2_, with the media refreshed every 2 days.

Immunofluorescence for NeuN and microtubule‐associated protein (MAP) after 5 days of culture, cells were fixed with 4% paraformaldehyde, permeabilized with 0.1% Triton‐X100, blocked with 5% BSA, incubated overnight at 4°C with rabbit anti‐NeuN (1:100, MAB11155, AmyJet Scientific) antibodies, then with Alexa Fluorconjugated seconday antibodies (1:200) for 2 h. DAPI was used for nuclear staining, and the cells were observed under a fluorescence microscope.

### 2.18 Assessment of Neuron Viability by CCK‐8 Assay

Cells were seeded into a 96‐well plate for 24 h. According to the kit's instructions, 10 μL of CCK‐8 solution was added per well, incubated for 4 h, and the absorbance values (OD value) at 450 nm were measured by microplate reader (Bio‐Rad, Hercules, CA, USA). Cell viability was calculated from standard curves to evaluate treatment effects.

### 2.19 Lactate Dehydrogenase (LDH) Assay

Neuronal damage was evaluated with LDH cytotoxicity assay kit (Beyotime, Shanghai, China). Briefly, 20 μL LDH was added 1 h before reoxygenation, cultured until OGD/R process. After centrifugation, 120 μL supernatant was collected, and absorbance at 490 nm was measured. Results were expressed as percentage relative to blank control group.

### 2.20 Statistical Analysis

All data were normalized before analysis. The one‐way analysis of variance (ANOVA) was used to compare multiple experimental groups, followed by Tukey's HSD test for post hoc analysis. Parametric data were presented as means ± standard deviation (SD), with significance set at *p* = 0.05 (*p* < 0.05 was considered statistically significant).

## 3 Results

### 3.1 The Protective Effects of XMZS on the Behavioral and Basic Brain Indexes of Mice With Cerebral Ischemia

**FIGURE 1:**
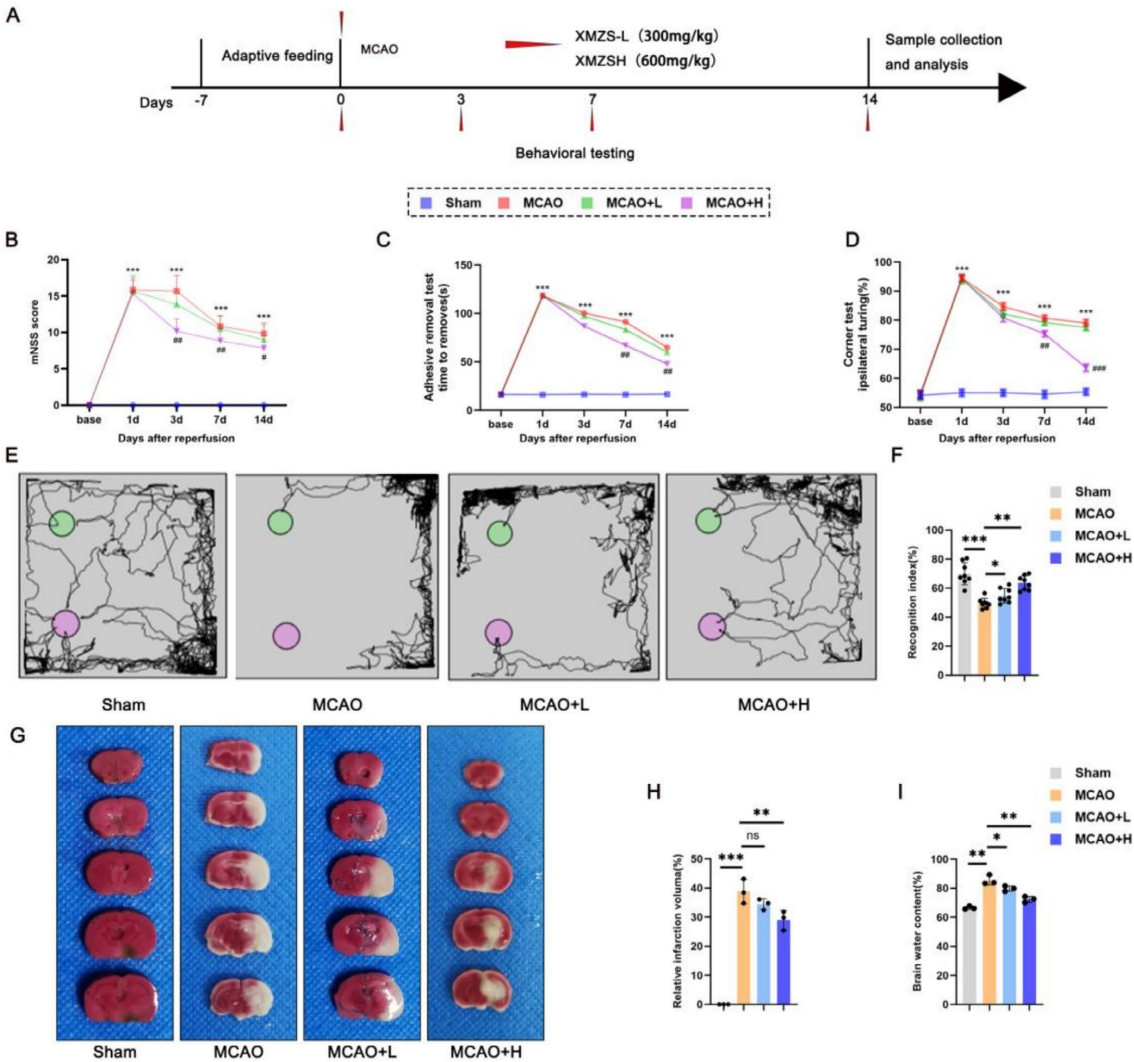
XMZS improves behavioral abnormalities and cerebral edema in mice with cerebral ischemia–reperfusion injury. (A) Experimental procedure. (B–D) Behavioral test results over time showing improvement in motor function and related parameters in different groups. (E, F) Visual representation (E) and quantitative analysis (F) of novel object recognition. (G) TTC‐stained brain slices of different treated groups. (H) Relative infarction volume. (I) Brain water content. *p < 0.05, **p < 0.01, ***p < 0.001, and ****p < 0.0001. The symbol “ns” represents no statistically significant difference between groups.

The experimental design is shown in Figure 1A. Behavioral tests (mNSS, Figure 1B; adhesive removal test, Figure 1C; corner test, Figure 1D) showed significant neurological deficits in MCAO group versus Sham group, while XMZS‐treated groups (MCAO + L and MCAO + H) improved over time, with high‐dose XMZS more effective. Novel object recognition test (Figure 1E,F) indicated that the mice in MCAO group had impaired function, which was inhibited by XMZS treatments, especially with the high‐dose. TTC staining (Figure 1G) Showed uniform red staining (no infarction) in Sham group, obvious white infarct regions in MCAP group. This showed that MCAO has successfully induced cerebral infarction. XMZS reduced infarct volume, with high‐dose more potent (Figure 1H). Brain water content (Figure 1I) was higher in MCAO group versus Sham group, while the XMZS‐treated groups had reduced infarct volumes, with the high‐dose XMZS group showing more significant reductions. These results suggested that high‐dose XMZS (63 g/kg/day) exhibits protective effects against cerebral ischemia–reperfusion injury in the MCAO mice model.

### 3.2 Changes in Immunohistochemical Indexes of Mice Based on XMZS Intervention From the Perspective of Microglia and Neurons

**FIGURE 2:**
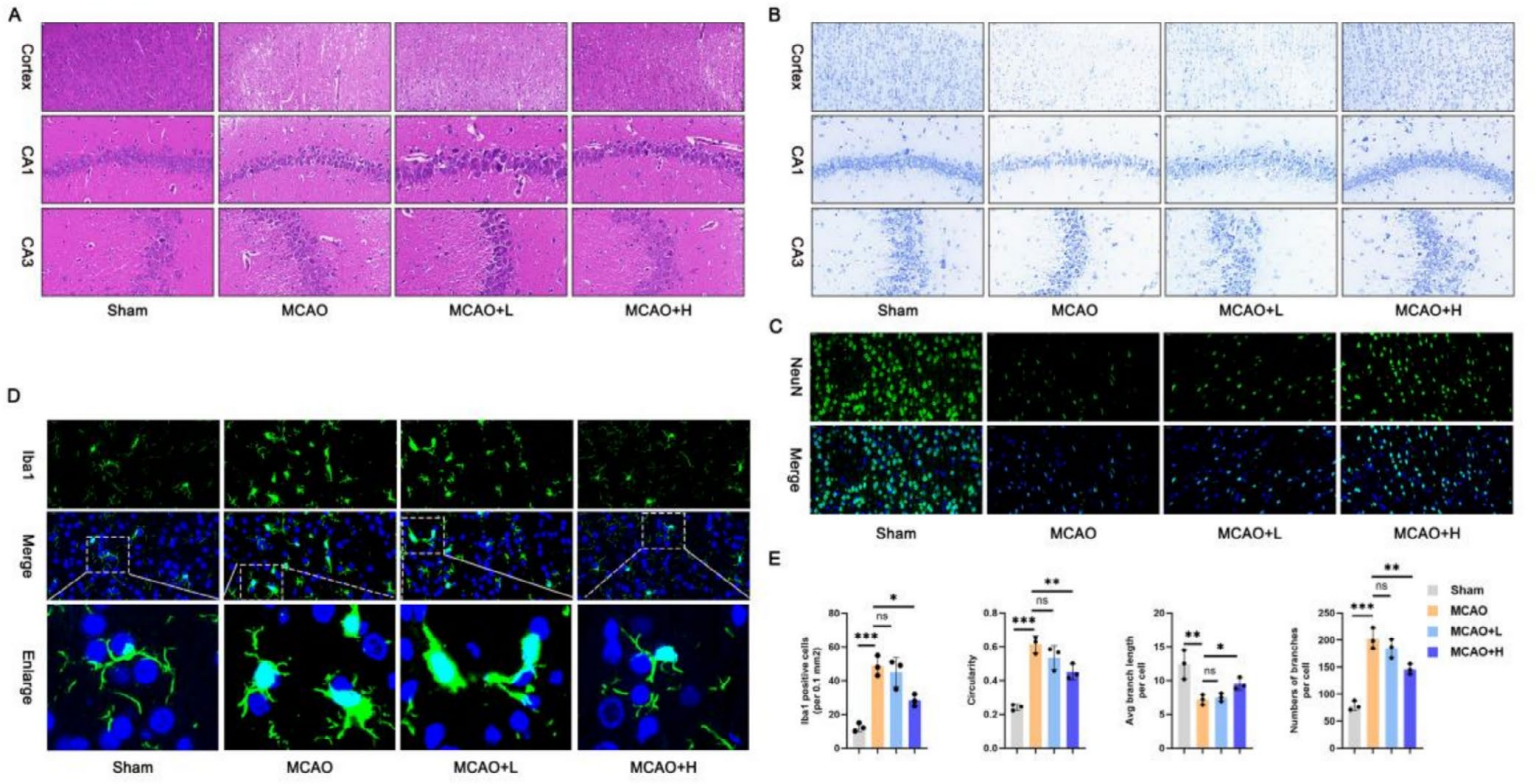
Microscopic and quantitative analysis of XMZS‐mediated improvement in brain tissue of different treated groups. (A) Brain injury detected by HE staining; (B) Protection of neurons detected by Nissl staining; (C, D) Immunofluorescence is utilized to detect the NeuN (C), and lba1 (D). (E) Quantitative analysis of microglia morphological features in different groups. *p < 0.05, **p < 0.01, ***p < 0.001, and ****p < 0.0001. The symbol “ns” represented no statistically significant difference between groups.

HE staining (Figure 2A) showed normal brain tissue morphology in the Sham group, severe damage (irregular cell shapes, discolored nuclei, and disrupted cytoplasmic integrity) in the MCAO group, and mitigated damage in the XMZS groups. Nissl staining (Figure 2B) revealed neuronal damage in the MCAO group with recovery in XMZS groups (high‐dose more effective). NeuN immunofluorescence (Figure 2C) showed reduced NeuN‐positive neurons in the MCAO group, increased in the XMZS groups, suggesting that the XMZS intervention can promote neuron survival (Figure 2C).

Iba1 immunofluorescence (Figure 2D) showed resting microglia (small cell bodies and long, ramified processes) in the Sham group, activated microglia (enlarged cell bodies and shorter, thicker processes) in the MCAO group, and resting tendency in the XMZS groups. Microglial analysis (Figure 2E) confirmed improved morphology in the XMZS groups. Overall, XMZS alleviates brain injury, protects neurons, inhibits microglial activation, and relieves inflammation and oxidative stress.

### 3.3 XMZS Alleviates Microglial Activation by Regulating the TLR4/MYD88/NF‐κB Signaling Pathway: Inflammatory Cytokines and Key Protein Expression in Mouse Blood and Tissues

**FIGURE 3:**
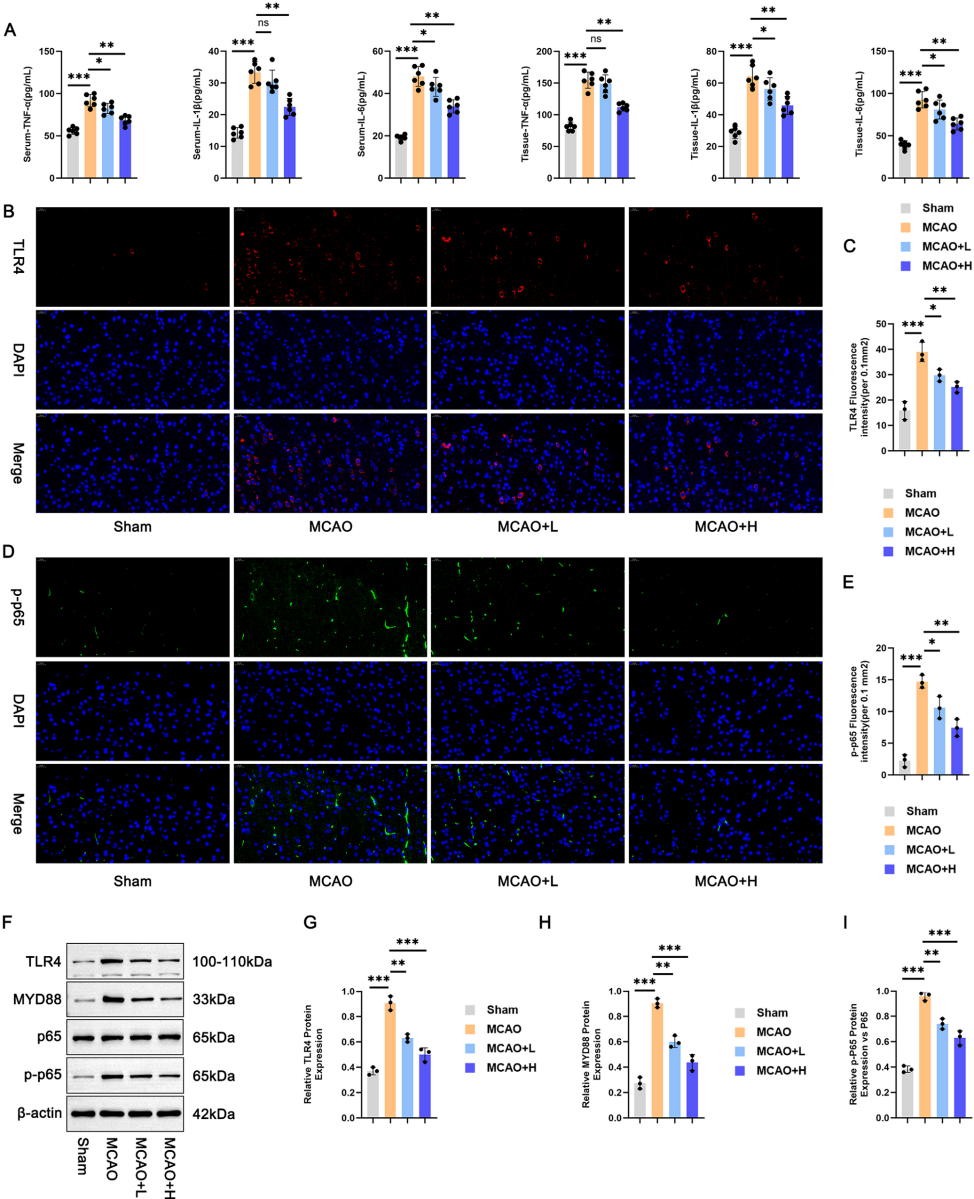
XMZS modulatory effects on inflammatory response and TLR4/MYD88/NF‐κB signaling pathway in cerebral ischemia–reperfusion injury. (A) ELISA results showing the levels of pro‐inflammatory cytokines (IL‐1β, IL‐6 and TNF‐α) in the serum and brain tissue. (B–E) Immunofluorescence images for observing the expression patterns of TLR4 and p‐p65. (F–I) Western blot analysis results for detecting the protein expression levels of TLR4, MYD88, p65 and p‐p65. *p < 0.05, **p < 0.01, ***p < 0.001, and ****p < 0.0001.

ELISA (Figure 3A) showed significantly increased IL‐1β, IL‐6, and TNF‐α in MCAO group plasma versus Sham group, decreased in XMZS groups, indicating inflammatory inhibition. Immunofluorescence (Figure 3B–E) showed enhanced TLR4 and p‐p65 fluorescence in the MCAO group, reduced in XMZS groups, confirming inhibited TLR4/NF‐κB pathway activation. Western blot analysis (Figure 3F–I) showed upregulated TLR4, MYD88, p65, and p‐p65 in MCAO group versus Sham group, downregulated in XMZS groups (total p65 stable). This suggested that XMZS targets multiple TLR4/MYD88/NF‐κB pathway nodes to alleviate inflammation.

### 3.4 In Vitro Level: Effects of XMZS‐Related Treatments on Key Proteins of Microglia

**FIGURE 4:**
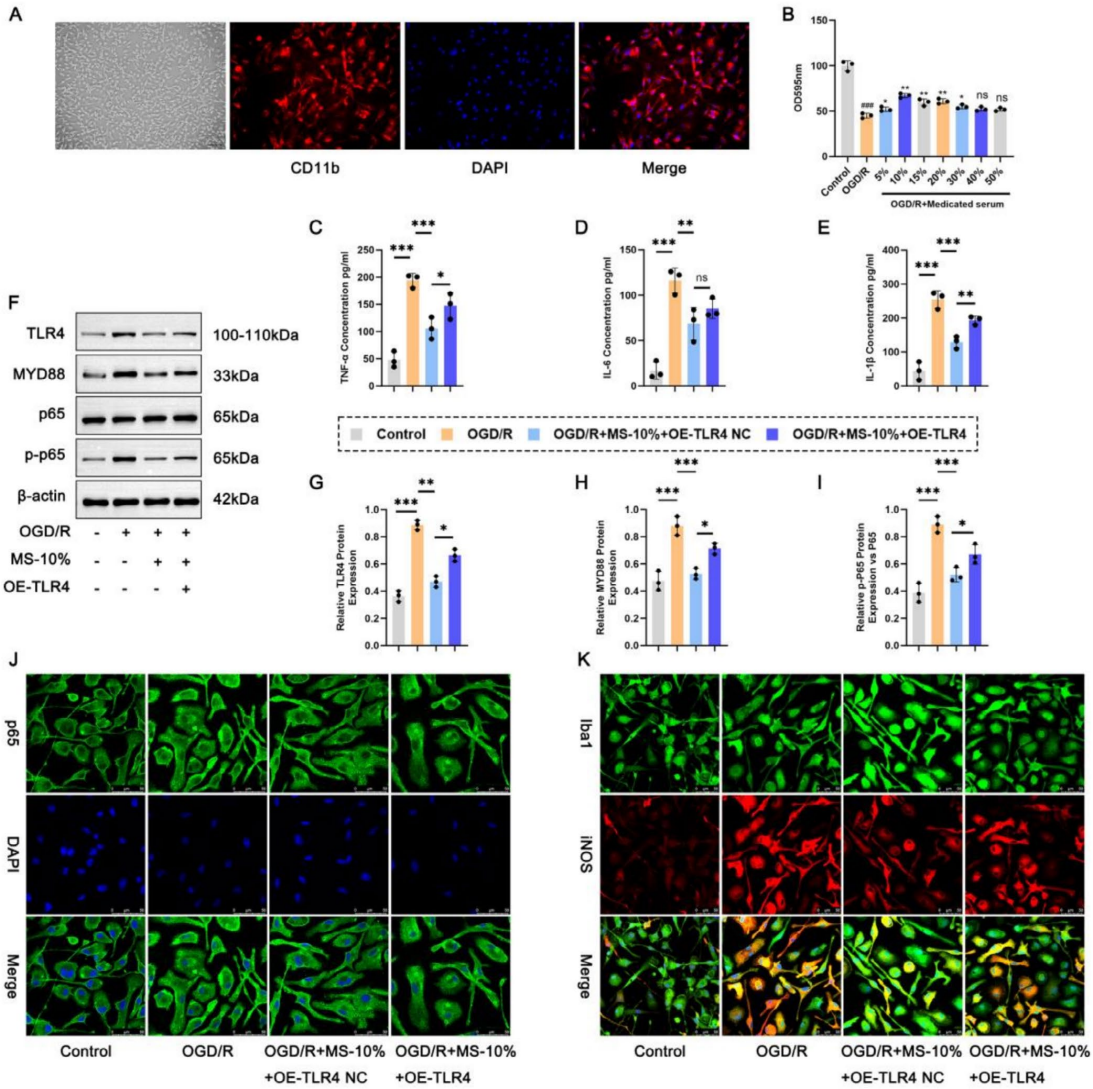
MS‐10% (with a concentration of 10% of XMZS in the serum) alters inflammatory response and TLR4/MYD88/NF‐κB pathway in microglial cells under oxygen–glucose deprivation/Reoxygenation (OGD/R) conditions. (A) Immunofluorescence staining images for CD11b, a marker for microglial cells. B: Effect of different plasma XMZS concentrations on cell viability detected by CCK8 assay; (C–E) ELISA results for detecting inflammatory cytokines (TNF‐α, IL‐6 concentration, IL‐1β). (F–I) Western blot analysis results for key components (TLR4, MYD88, p65, and p‐p65) of the TLR4/MYD88/NF‐κB pathway. (J) Immunofluorescence images showing the nuclear translocation of p65, a key step in NF‐κB pathway activation. (K) Immunofluorescence images showing the expression of lba1 (a microglial activation‐associated enzyme) and iNOS (an inflammation‐associated enzyme). *p < 0.05, **p < 0.01, ***p < 0.001, and ****p < 0.0001. The symbol “ns” represented no statistically significant difference between groups.

Primary microglia were isolated, and cell groups were constructed. CD11b immunofluorescence (Figure 4A) and quantification (Figure 4B) confirmed successful microglial isolation. ELISA (Figure 4C–E) showed increased inflammatory cytokines in the OGD/R group versus Control group, reduced by MS‐10%, indicating anti‐inflammatory activity. Western blot analysis (Figure 4F–I) showed up‐regulated TLR4/MYD88/NF‐κB pathway proteins in OGD/*R* + MS‐10% + OE‐TLR4, revealing TLR4 overexpression interaction with MS‐10%. Immunofluorescence (Figure 4J) showed increased p65 nuclear translocation in the OGD/R group, reduced in treatment groups. Iba1 and iNOS expression (Figure 4K) was high in the OGD/R group, suppressed by MS‐10% but partially offset by TLR4 overexpression. These results confirm MS‐10% modulates TLR4/MYD88/NF‐κB pathway and microglial activation in OGD/R injury.

### 3.5 In Vitro Culture of Neurons: The Role of the XMZS Intervention in the Survival and Apoptosis of Neurons

**FIGURE 5:**
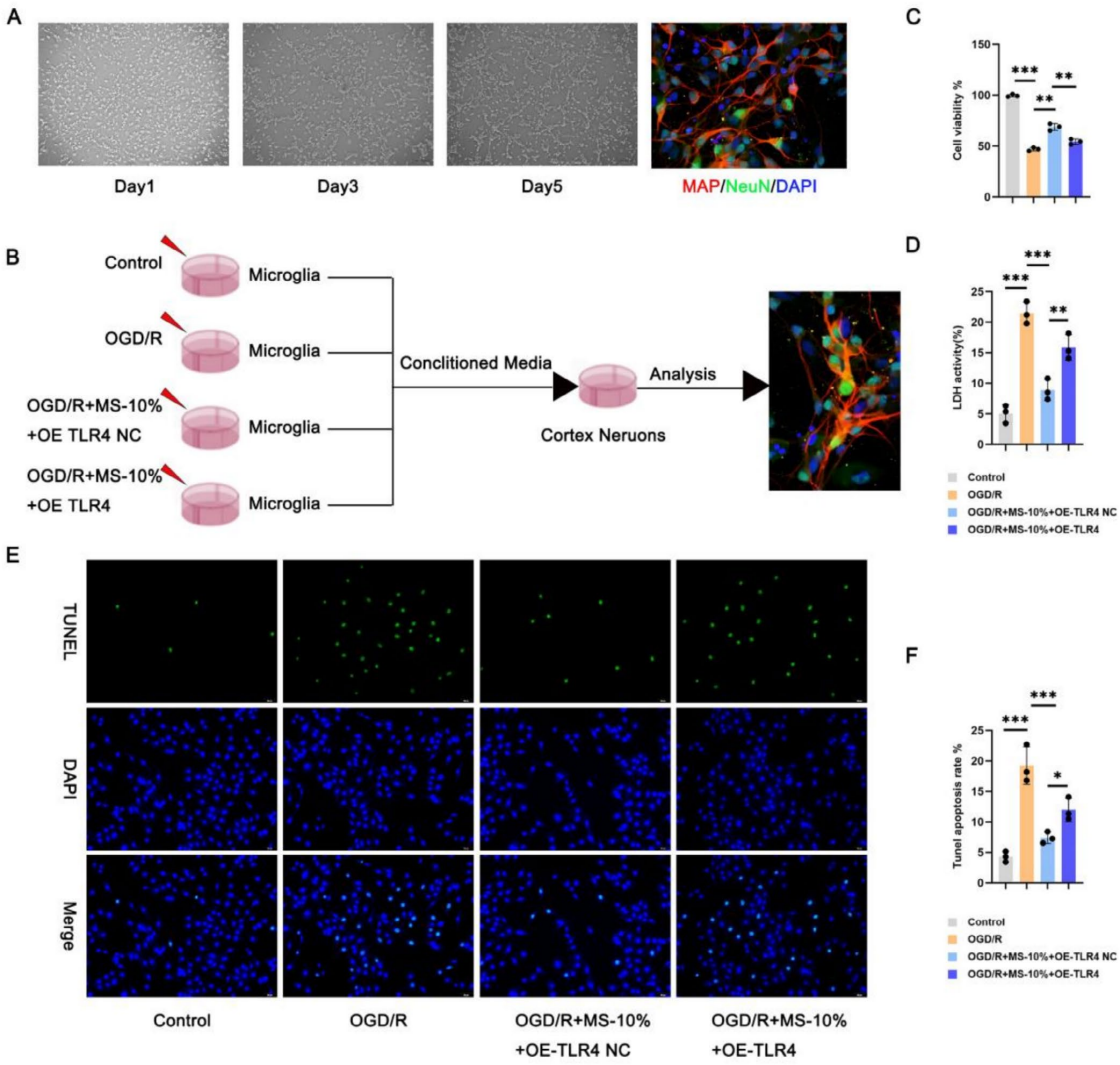
Neuroprotective effects of MS‐10% on neurons under OGD/R: Restoring function, promoting survival, and modulating signaling pathways. (A) Isolation and identification of primary neurons. (B) Treatment group setup for microglia neuron co‐culture study. (C) Cell viability assessed by CCK8. (D) LDH activity showing the membrane integrity and survival of neurons. (E, F) TUNNEL staining images and quantitative data offering a comprehensive view of cortical neuron apoptosis. *p < 0.05, **p < 0.01, ***p < 0.001, and ****p < 0.0001.

Cortex neurons were cultured in microglial conditioned media. Immunofluorescence (Figure 5A) showed neuron growth over 5 days. Experimental design is shown in Figure 5B. CCK8 assay (Figure 5C) showed decreased viability in OGD/R group, recovered by MS‐10% (more obvious with OE‐TLR4 NC). LDH assay (Figure 5D) showed elevated LDH in OGD/R group, reduced by MS‐10% + OE‐TLR4 NC, with partial reversal by TLR4 overexpression. TUNEL staining (Figure 5E,F) showed high apoptosis in OGD/R group, inhibited by MS‐10%, with TLR4 overexpression counteracting this effect.

### 3.6 In Vivo Verification: Re‐Exploration of the Protective Efficacy of XMZS on the Behavioral and Brain Indexes of Mice With MCAO

**FIGURE 6:**
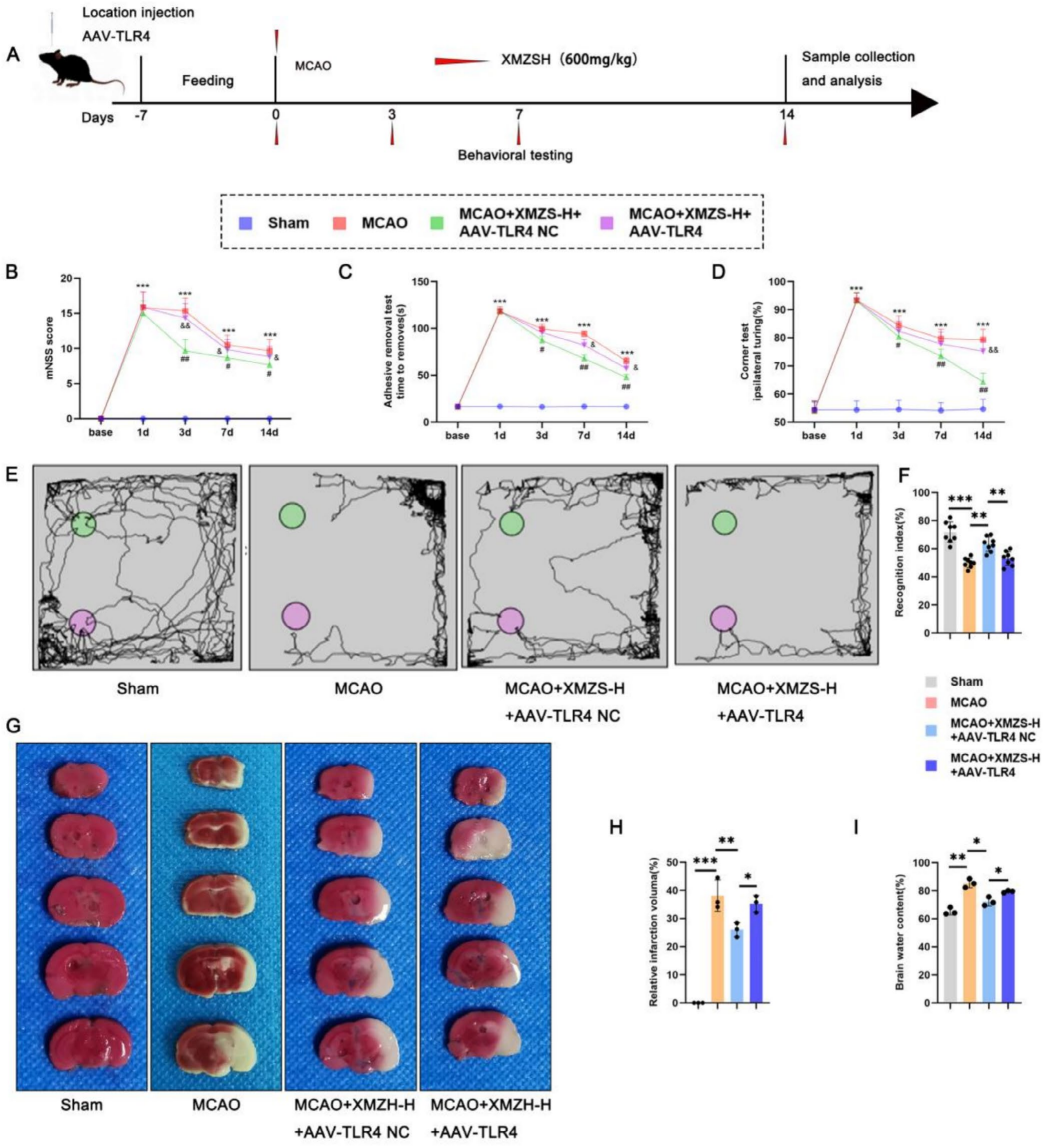
The impact of XMZS in MCAO mice with AAV‐TLR4 transfection: Behavioral, cognitive, and infarct volume analysis. (A) Experimental timeline for AAV‐TLR4 injection, MCAO induction, and XMZS administration in mice. (B–D) The results of behavioral test on mNSS score (B), adhesive removal test (C), corner test (D). (E, F) Novel object recognition test results. (G, H) Assessment of cerebral infarction volume; (I) Brian water content. *p < 0.05, **p < 0.01, ***p < 0.001, and ****p < 0.0001.

Mice were injected with AAV‐TLR4 on Day 7, subjected to MCAO on Day 3, and treated with high‐dose XMZS (63 g/kg) from Day 7 (Figure 6A). Behavioral tests (Figure 6B–D) showed normal neurological function in the Sham group, severe impairment in the MCAO group, improved by XMZS (more effective with AAV‐TLR4 NC). Novel object recognition test (Figure 6E,F) showed reduced recognition index in MCAO group, increased by XMZS, with TLR4 overexpression attenuating this effect. Infarction volume (Figure 6G–I) showed no infarction in the Sham group, an obvious infarct in the MCAO group, reduced by XMZS (more significantly with AAV‐TLR4 NC), confirming TLR4 overexpression weakens XMZS's infarct‐reducing effect.

### 3.7 In Vivo Verification: Re‐Exploration of the Protective Efficacy of XMZS on the Morphology and Inflammatory Cytokine Levels

**FIGURE 7:**
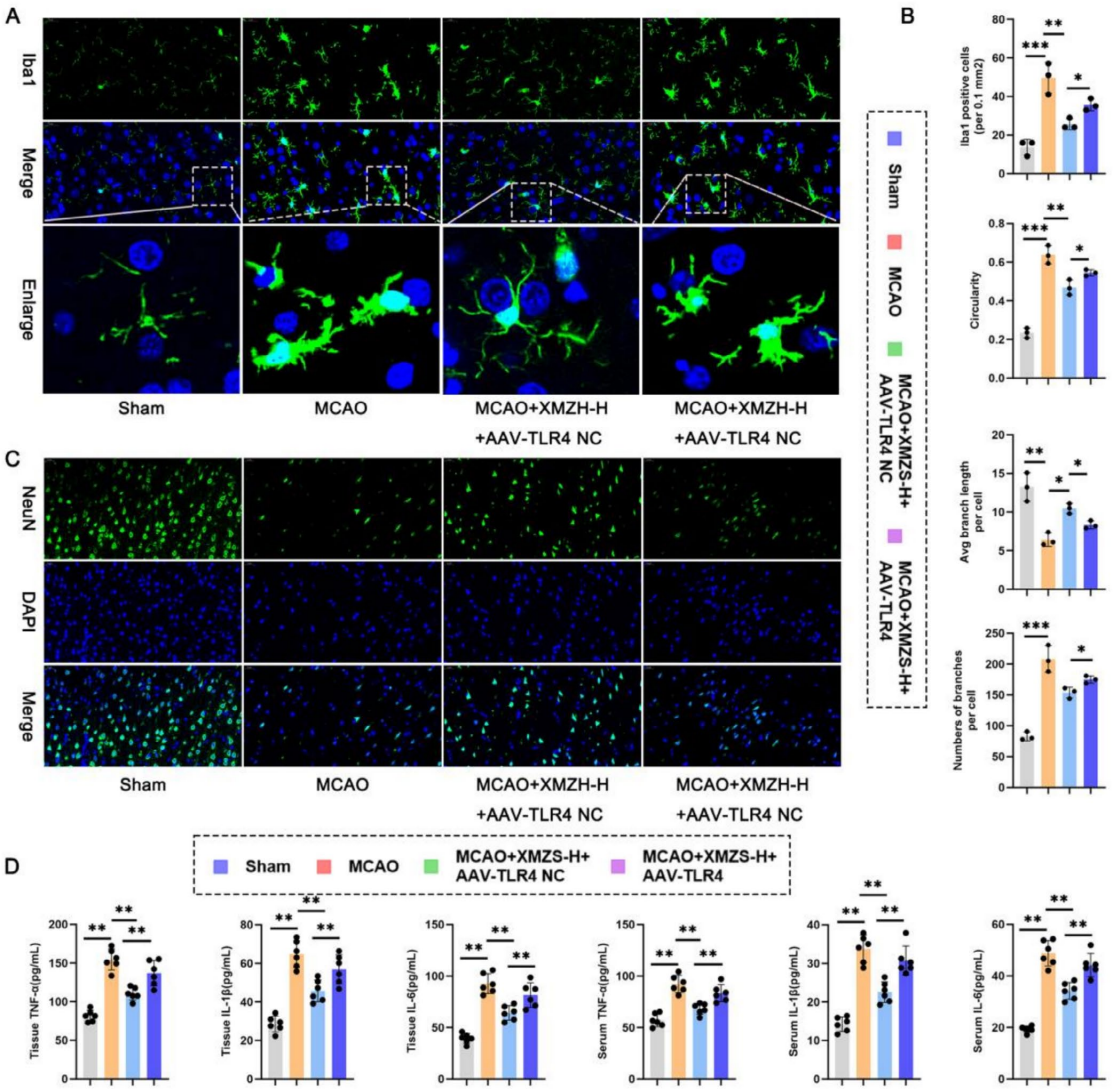
The impact of XMZS and TLR4 overexpression on microglial activation, neuronal status, and inflammatory cytokine levels in MCAO model. (A) Immunofluorescence visualization of lba1; (B) Quantitative analysis of lba1 and microglia morphological features in different groups. (C) Immunofluorescence visualization of NeuN. (D) Levels of inflammatory cytokines in plasma and tissues. *p < 0.05, **p < 0.01, ***p < 0.001, and ****p < 0.0001.

Immunofluorescence (Figure 7A,C) showed resting microglia and intact neurons in Sham group, activated microglia and reduced neurons in the MCAO group, improved by XMZS but exacerbated by TLR4 overexpression. Quantitative analysis (Figure 7B,D) confirmed activated microglial characteristics (reduced branch/length, increased circularity) in MCAO group, reversed by XMZS, counteracted by TLR4 overexpression. Inflammatory cytokines (IL‐1β, IL‐6, TNF‐α) were elevated in MCAO group, reduced by XMZS, rebounded by TLR4 overexpression. Thus, XMZS mitigates microglial activation and protects neurons via TLR4 inhibition.

## 4 Discussion

Stroke‐induced neuroinflammation, driven by microglial overactivation via the TLR4/MYD88/NF‐κB signaling pathway, represents a critical therapeutic target for mitigating secondary brain injury. In this study, we investigated the effect of XMZS on microglial‐mediated neuroinflammation in a mouse model of cerebral ischemia‐refusion injury and explored its underlying molecular mechanisms. The results demonstrated that XMZS, especially at a high dose (63 g/kg), exerted significant protective effects against cerebral ischemia–reperfusion injury, primarily by suppressing TLR4‐mediated inflammatory cascades. Our findings showed that XMZS treatment significantly improved neurological and cognitive functions, reduced infarct volume, and alleviated brain edema. These effects were accompanied by notable decrease in pro‐inflammatory microglial activation, evidenced by reduced co‐localization of lba1 + iNOS and reduction of inflammatory cytokines (IL‐1β, IL‐6, TNF‐α) in both brain tissues and in vitro microglial cultures. These outcomes were supported by XMZS's ability to disrupts the TLR4/MYD88/NF‐κB pathway at multiple levels: down‐regulating TLR4 protein expression, inhibiting MYD88 expression and its interaction with TLR4, and blocking the nuclear translocation of phosphorylated p65. These findings were validated through the western blotting, co‐immunoprecipitation, and confocal microscopy. The following rescue experiments using AAV9‐mediated TLR4 overexpression in the microglial cells, suppressed XMZS‐induced protection, restoring pro‐inflammatory cytokine production and NF‐κB activation, thus confirming TLR4 as a direct and essential target of XMZS’ action.

The neuroprotection conferred by XMZS was closely associated with its modulation of microglial phenotype. M1‐type microglia, activated in a classical manner, trigger a defensive response against brain injury and are pro‐inflammatory. Studies have shown that the activation of microglia and the release of pro‐inflammatory cytokines like IL‐1β, IL‐6, TNF‐α play a key role in the neuroinflammation and subsequent neural damage after stroke (Xie et al. 2024; Chen et al. 2017). On the other hand, M2‐type microglia, which are alternatively activated, usually offer neuroprotective effects. They secrete anti‐inflammatory cytokines such as IL‐4, IL‐10, and TNF‐β, etc. (Wu, Dong, et al. 2023). Our results demonstrated that the conditioned medium for MXZS‐treated microglia significantly increased the primary neuron viability, indicating that suppressed microglial inflammation directly contributes to neuronal survival. Given the role of M1 microglia in promoting inflammation and potential harm to neurons, it is highly likely that XMZS exerts its effect by modulating the M1/M2 microglial balance. Specifically, it may suppress the activation of M1 microglia, reducing the release of those harmful proinflammatory cytokines. Consequently, the MXZS alleviates the inflammatory stress on neurons, thereby contributing to neuronal survival.

Importantly, the observations from in vitro experiments were consistent with the findings from in vivo experiments. XMZS could specifically reduce the expression level of TLR4 in lba1‐positive microglia within the peri‐infarct region. TLR4 is a key receptor in the signaling pathway that activates M1 microglia (Yang et al. 2021). By reducing this expression, XMZS interrupts the “ignition switch” for M1 microglial overactivation. This establishes a cell‐type‐specific mechanism, indicating that the XMZS targets M1 microglia precisely in the context of the pre‐infarct region. It not only helps to mitigate the harmful effects of M1‐mediated neuroinflammation, but also potentially facilitates the shift towards a more beneficial M2‐dominant microglial state, thus playing a pivotal role in promoting neuroprotection and neural recovery after stroke.

A key contribution of this study lies in the detailed dissection of the TLR4/MYD88/NF‐κB signaling cascade, revealing XMZS as a multi‐component inhibitor acting on both upstream and downstream nodes. This pathway is intricately involved in the inflammatory cascade subsequent to cerebral ischemia–reperfusion injury (Rahimifard et al. 2017). Activation of TLR4 upon recognition of pathogen‐associated molecular patterns (PAMPs) or damage‐associated molecular patterns (DAMPs) initiates a signaling cascade through MYD88, ultimately leading to the activation of NF‐κB (Wang et al. 2024). Once activated, NF‐κB translocates to the nucleus and promotes the transcription of pro‐inflammatory cytokines such as TNF‐α, IL‐β, and IL‐6, which exacerbate neuroinflammation and tissue damage (Wu, Tan, et al. 2023). In the context of our study on XMZS, several lines of evidence suggest its potential interaction with the TLR4/MYD88/NF‐κB pathway. Experimental investigations have demonstrated that XMZS can modulate the expression levels of key components of this pathway. In both in vitro cell models and in vivo mice models of cerebral ischemia–reperfusion, XMZS treatment has been shown to down‐regulate the expression of TLR4, MYD88, and NF‐κB p65. This down‐regulation is accompanied by a significant reduction in the production and release of pro‐inflammatory cytokines, indicating that XMZS exerts an anti‐inflammatory effect by suppressing the TLR4/MYD88/NF‐κB signaling pathway.

One possible mechanism underlying this regulatory effect of XMZS could be its direct interaction with TLR4. It is hypothesized that certain bioactive components within XMZS may bind to TLR4, either competing with its natural ligands or altering its conformation, thereby preventing its activation and subsequent signaling. Additionally, XMZS may also influence the downstream signaling events by interfering with the interaction between MYD88 and TLR4 or by modulating the phosphorylation and activation of NF‐κB. Furthermore, XMZS may exert its effects on the TLR4/MYD88/NF‐κB through indirect mechanisms. It has been reported that XMZS possesses antioxidant properties (such as *Paeonia lactiflora* Pall. (Goto et al. 2015), *Angelica sinensis* (Li et al. 2023), Radix *Ginseng* (Hao et al. 2024), and *Rehmannia glutinosa* Libosch. (Ren et al. 2023)), and oxidative stress is known to play a crucial role in the activation of the TLR4 pathway (Lisboa et al. 2024). By scavenging free radicals and reducing oxidative stress, XMZS may indirectly inhibit the activation of TLR4 and subsequent downstream signaling. Moreover, XMZS may also interact with other signaling pathways that cross‐talk with the TLR4/MYD88/NF‐κB pathway to modulate the overall inflammatory response.

The modulation of the TLR4/MYD88/NF‐κB pathway by XMZS has significant implications for the treatment of ischemic stroke and associated coronary artery injuries. By suppressing the over‐activation of this pathway, XMZS can reduce neuroinflammation, protect neuronal cells from damage, and improve neurological outcomes.

Additionally, the precise molecular mechanisms by which XMZS interacts with this pathway, especially at the atomic and molecular levels, require more in‐depth investigation. Future studies could also explore the long‐term effects of XNZS treatment on the TLR4/MYD88/NF‐κB pathway and its impact on the recovery and prognosis of patients with ischemic stroke. Furthermore, the key bioactive components of XMZS and the synergistic interactions between components underlying its therapeutic effects warrant additional research.

In conclusion, our study provides compelling evidence for the role of XMZS in modulating the TLR4/MYD88/NF‐κB signaling pathway, which may underlie its therapeutic effects in treating ischemic stroke injury. This finding not only deepens our understanding of the pharmacological mechanism of XMZS but also offers a new perspective for the development of novel therapeutic strategies for cerebrovascular disease.

## Author Contributions

**Xi Liu:** writing – original draft. **Hanchang Yu:** validation. **Xiyun Fei:** formal analysis. **Zhijun Zhong:** writing – review and editing, conceptualization. **Zhongxu Hu:** visualization. **Cheng Wang:** methodology, data curation. **Min Peng:** visualization. **Yi'an Xiao:** data curation. **Ling Han:** software. **Jilin Zhou:** validation.

## Funding

This research was supported by the Natural Science Foundation of Hunan Province (Grant 2024JJ9527); the Traditional Chinese Medicine Research Project of Hunan Province (Grant C2024029); the Natural Science Foundation of Changsha City (Grant kq2502318); and the Scientific Research Project of the Changsha Municipal Health Commission (Grant KJ‐B2023080).

## Supporting information


**Table S1:** Composition of Xuming Zhusan (XMZS) herbal formula.

## Data Availability

The data used to support the findings of this study are available from the corresponding author upon reasonable request.
