# Supplementary material for: Xuming Zhusan Decoction Attenuates Post‐Stroke via Modulating TLR4/MYD88/NF‐κB Pathway in Mice
Source: Food Sci Nutr. 2026 Apr 16;14(4):e71734. doi: 10.1002/fsn3.71734 (PMC13087087; doi:10.1002/fsn3.71734)
Supplement: Supplementary file 1 — Table S1: Composition of Xuming Zhusan (XMZS) herbal formula. [file FSN3-14-e71734-s001.docx]

**Table S1 Composition of Xuming Zhusan (XMZS) herbal formula**

| Latin Binomial | Dosage (g) |
| --- | --- |
| *Pueraria lobata* (Willd Ohwi) | 30 |
| *Heracleum hemsleyanum* Diels | 15 |
| *Paeonia lactiflora* Pall. | 15 |
| *Angelica sinensis* | 15 |
| *Cinnamomum cassia* Presl | 12 |
| *Polygala tenuifolia* Willd. | 12 |
| Radix *Ginseng* | 10 |
| *Saposhnikovia divaricata* | 10 |
| *Rehmannia glutinosa* Libosch. | 10 |
| *Ligusticum chuanxiong* | 9 |
| *Ephedra sinica* Stapf | 9 |
| *Asarum heterotropoides* F. Schmidt | 6 |

Note: Composition of Xuming Zhusan (XMZS) herbal formula. The total weight of the 12 herbs is 100 g, and the dosage of each herb corresponds to the formula ratio used in this study.
